# Consensus on measurement properties and feasibility of performance tests for the exercise and sport sciences: a Delphi study

**DOI:** 10.1186/s40798-016-0071-y

**Published:** 2017-01-05

**Authors:** Sam Robertson, Peter Kremer, Brad Aisbett, Jacqueline Tran, Ester Cerin

**Affiliations:** 1 Centre for Exercise and Sport Science, Deakin University, Geelong, Victoria Australia; 2 Institute of Sport Exercise and Active Living (ISEAL), Victoria University, Footscray Park campus, PO Box 14428, Melbourne, Victoria 8001 Australia; 3 Centre for Physical Activity and Nutrition Research, Deakin University, Burwood, Victoria Australia; 4 School of Public Health, The University of Hong Kong, Hong Kong, China

**Keywords:** Performance tests, Sports testing, Reliability, Validity, Responsiveness, Assessment, Delphi

## Abstract

**Background:**

Performance tests are used for multiple purposes in exercise and sport science. Ensuring that a test displays an appropriate level of measurement properties for use within a population is important to ensure confidence in test findings.

The aim of this study was to obtain subject matter expert consensus on the measurement and feasibility properties that should be considered for performance tests used in the exercise and sport sciences and how these should be defined. This information was used to develop a checklist for broader dissemination.

**Methods:**

A two-round Delphi study was undertaken including 33 exercise scientists, academics and sport scientists. Participants were asked to rate the importance of a range of measurement properties relevant to performance tests in exercise and sport science. Responses were obtained in binary and Likert-scale formats, with consensus defined as achieving 67% agreement on each question.

**Results:**

Consensus was reached on definitions and terminology for all items. Ten level 1 items (those that achieved consensus on all four questions) and nine level 2 items (those achieving consensus on ≥2 questions) were included. Both levels were included in the final checklist.

**Conclusions:**

The checklist developed from this study can be used to inform decision-making and test selection for practitioners and researchers in the exercise and sport sciences. This can facilitate knowledge sharing and performance comparisons across sub-disciplines, thereby improving existing field practice and research methodological quality.

## Key points

- Traditional measurement properties such as re-test reliability, rater reliability, content validity and discriminant validity were agreed to be important in all applications.
- Items not commonly considered in the literature as integral to performance tests were also agreed upon as important, including test feasibility, interpretability and duration.
- The checklist developed in this study can be used to inform performance test development or selection by exercise and sport scientists.

## Background

In the exercise and sport sciences, a performance test can be defined as a measurement or series of measurements that help to determine the health status, physical fitness or sport-specific ability of an individual [1–4]. Performance tests serve a variety of purposes in exercise and sport scenarios, including assessing the effectiveness of researcher- or practitioner-implemented interventions [5–7] or monitoring participant progress within a prescribed exercise programme [3, 8]. These tests can also be used to gather objective evidence of a participant’s strengths and weaknesses [9, 10] or provide diagnostic information relating to the health (e.g., injury) status of an individual [3, 11, 12]. In sport, performance tests are also used to inform the identification and selection of talented young participants, which may assist governing bodies when prioritising the time and financial contributions they invest into their athletes [13, 14].

The importance of physical performance tests displaying adequate measurement properties has received considerable attention in the exercise and sports science literature. Measurement properties such as reliability [1, 4, 15, 16], validity [1, 17] and responsiveness [1, 4, 18] have all been investigated with respect to their importance. Various industry and governing bodies have also published ethical guidelines for the undertaking of such testing [19, 20]. Despite this, no specific recommendations or guidelines exist to inform the selection or the design of a performance test based on its measurement properties and feasibility. This is an important consideration on many levels. For instance, multiple tests are often available to measure the same performance construct, each with their own relative strengths and limitations. For example, maximal oxygen uptake during exercise can be estimated using running [21, 22], walking [23], step [24] and cycling-based [24] tests. Such tests can also vary in both their content and purpose (i.e., to discriminate participants, identify talent or assess the effect of an intervention). The use of an unsuitable test may lead to a variety of adverse consequences. These could include a risk of incorrect conclusions being reached on an individual’s physical status, increased assessment error, non-evidence-based practice and a lack of comparative data across exercise disciplines, sports or research studies. In the worst case scenario, it may also mean that clients, patients, research participants and athletes are put at risk during testing procedures.

Similar problems have recently been addressed in other disciplines, most notably medicine [25], health [26] and physical activity [27]. However, despite the success of these approaches for their target disciplines, the large number of redundant items in these studies with respect to exercise and sport science means that their direct application may not be appropriate. As an example, previous manuals developed in health, rate questionnaires on their cross-cultural validity and the ability to be translated into multiple languages [28], which may be less relevant for many of the exercise and sport sciences.

Previous research has utilised the Delphi technique to obtain the consensus needed by content experts in order to develop such a set of standardised guidelines [28–30]. The Delphi approach utilises groups of subject matter experts responding anonymously to a series of subsequent questionnaires, with repeated feedback used in order to reach consensus among the group [31, 32]. Recent work in other disciplines has successfully undertaken this task (e.g. quality of life research [28, 33], medicine [34] and nursing [29]) developing user-friendly and easily applicable checklists based on the resulting findings [33]. For example, publications from the COSMIN framework (a manual developed to help assess the measurement properties of health reporting questionnaires) have experienced considerable citations (over 500) in the 5 years since its 2010 inception [26, 28, 35, 36]. In order to improve physical performance testing quality and quality control, along with standardisation of test selection (thereby allowing comparison across sub-disciplines), a specific framework for use in exercise and sport sciences is needed.

The primary aim of this study was to obtain subject matter expert consensus on which measurement and feasibility properties should be considered for performance tests used in the exercise and sport sciences, as well as how these should be defined. A secondary aim was to develop a checklist which can be implemented to inform performance test development or selection.

## Methods

### Participants

Three types of exercise and sport scientists were recruited for participation in this Delphi study. These were defined as (i) clinical exercise scientists/exercise physiologists, (ii) sport scientists and (iii) academics. These groups were specifically targeted for inclusion in the study given their potential application of the findings in their work environment. Participants may have fit more than one category in some instances, however indicated their ‘primary’ affiliation at the start of the survey process. Data collection was undertaken via internet-based questionnaires, with the exact number of rounds dependent on the rate with which consensus on specific items was achieved.

Participants were recruited via methods shown previously to produce highest response rates in Delphi studies [37], including contacting personal industry contacts and cold contacting via publicly provided email addresses. Inclusion criteria for participants were set as follows. Clinical exercise scientists/exercise physiologists were required to (a) maintain current accreditation with their relevant accrediting body and (b) have ≥5 years’ experience in the clinical exercise or exercise physiology industry. Sport scientists were required to be currently employed by a professional sporting club or institution. Those undertaking senior management roles were specifically targeted. For academics, a background of publications relating to measurement properties of testing in exercise or sport (≥3 articles) was required.

Although a variety of methods can be used when implementing Delphi studies [28], it is preferable for a minimum of 15 subject matter experts to be recruited [32, 38]. In accounting for potential non-response, a total of 136 individuals were contacted for participation in the Delphi procedure. Of these, 13 declined to participate, 90 did not respond and 33 (24% of total invited) agreed to participate. Following provision of written consent, panel members provided an information letter via email outlining specific details relating to the overall aims, study procedures and requirements of the study.

### Delphi study—round 1

**Fig. 1:**
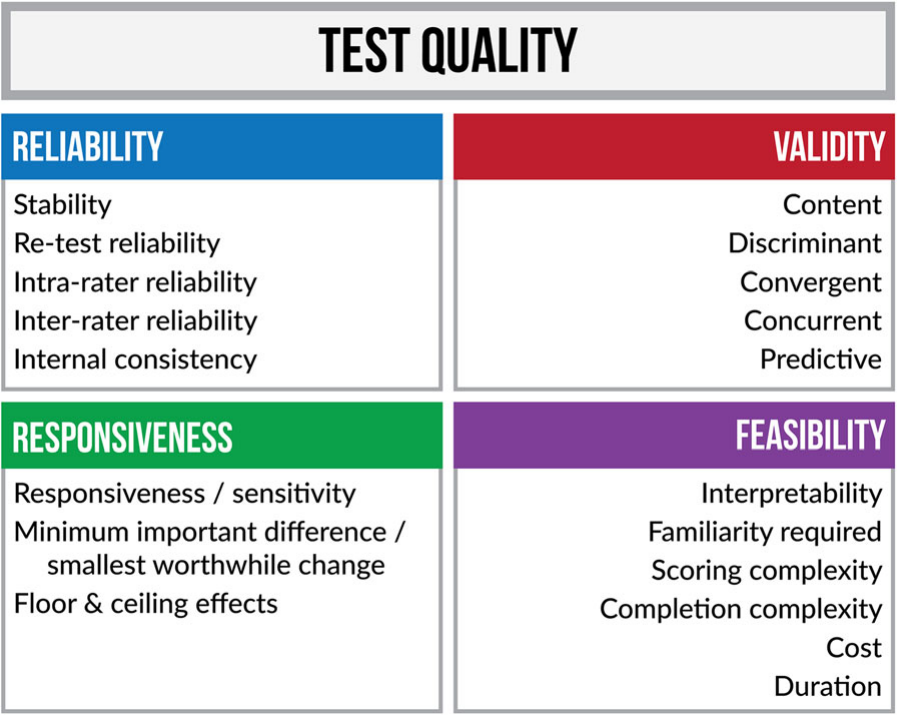
Taxonomy including the initial measurement properties and feasibility as sent to participants as part of the first Delphi round

All rounds of the Delphi questionnaire were developed and administered to participants via a commercial survey provider (SurveyMonkey Inc, CA, USA). Prior to the first Delphi round, a steering committee was created [29], comprising all five authors. Information relating to physical performance test measurement properties and their definitions were primarily developed by the first author, with revisions made based on feedback from the steering committee. The questionnaire items were based upon content extracted from previous systematic reviews undertaken in exercise and sport science, as well as other disciplines. Once finalised, the initial taxonomy administered to participants grouped the 20 items into four distinct categories (Fig. 1). All participants were presented with this list (including reference support for each item) which also included a range of questions relating to each item.

Specifically, for each item included in the first round of the questionnaire, participants were asked (i) whether it should be considered when evaluating the quality of a performance test and (ii) whether they agreed with the terminology and definition used to describe the item. In interpreting the latter question, ‘terminology’ referred to the name of the item itself, whereas ‘definition’ comprised the explanation assigned to each item. Additionally, using a 5-point Likert scale, participants were also asked to (iii) rate the importance of each item for determining the quality of an exercise and sports performance test, with 1 indicating ‘not at all important’ and 5 considered ‘extremely important’. The final question for each item also required a second 5-point Likert response, asking (iv) the participant’s attitude to the item with 1 indicating ‘I never consider this item’ and 5 indicating ‘I always consider this item’. The Likert scale responses were used to guard against information loss which can occur when experiencing response dichotomisation [39]. For all four questions, participants were permitted to provide explanation and/or justification for their response using a text box inserted into the questionnaire.

The questionnaire was left open for 15 weeks in order to facilitate the largest response rate possible. Following this, all participant responses were exported for further analysis. Based on the summation of scores received, all items were then ranked by the steering committee. Newly suggested items recommended for addition to the list were arranged in order of the frequency with which they were suggested. In order for an item to achieve consensus, a minimum of 67% agreement was required from the participants with respect to the four questions [28, 32]. For the two Likert-scale responses, this constituted a minimum of 67% of participants rating the item as 4 or 5 for both questions.

### Delphi questionnaire—round 2

In the second Delphi round, participants received (i) a summary of results from the previous round, (ii) a transcript of written responses from other participants, and (iii) a list of proposed revisions as determined by the steering committee based on these responses. Each participant also received a link to the next round of the questionnaire. At this point, participants were asked to provide a binary ‘yes’ or ‘no’ response to each proposed revision, whilst also being able to consider the responses from other participants and results from the preceding round [32].

## Results

### Participants

Of the 33 individuals that provided consent for participation in the study, a total of 28 (response rate (RR) 21% of total approached, RR 85% of those who agreed and consented) provided responses to round 1. The 28 participants consisted of 14 academics, eight sport scientists and six clinical exercise scientists/exercise physiologists. The names of all panel members who completed at least one round are presented in the “Acknowledgements” section.

### Round 1

Results relating to the four questions asked of each item are shown in Table 1. All 20 items proposed in the initial questionnaire achieved a minimum 67% consensus with respect to whether the item should be considered (range = 68 to 100%), whilst terminology and definitions reported similar levels of agreement (68 to 100%). Of the 20 items, 10 also achieved consensus with respect to the participants’ rating of item importance and attitude (via the Likert-scale responses).

**Table 1:** Results relating to round 1 of the Delphi study, including specific percentage of consensus reached for each of the four questions

		Q1	Q2	Q3		Q4	
Group	Item	Consider the item?	Definition and terminology	Importance to quality (mean)	% responses level 4 or 5	Attitude to item (mean)	% responses level 4 or 5
Reproducibility/reliability	Stability	71.4	68.2	3.62	65.4^a^	3.62	69.2
	Re-test reliability	92.9	96.0	4.5	85.7	4.43	85.7
	Intra-rater	100.0	92.9	4.5	92.9	4.46	89.3
	Inter-rater	100.0	92.9	4.46	89.3	4.5	89.3
	Internal consistency	67.9	100.0	3.39	50.0^a^	3.29	46.4^a^
Validity	Content validity	100.0	89.3	4.68	96.4	4.64	96.4
	Discriminant validity	100.0	92.9	4.21	82.1	4.14	75.0
	Convergent validity	78.6	91.3	3.14	28.6^a^	3.11	28.6^a^
	Concurrent validity	82.1	88.9	3.25	32.1^a^	3.25	35.7^a^
	Predictive validity	85.7	91.7	3.79	64.3^a^	3.71	60.7^a^
Responsiveness	Responsiveness	100.0	89.3	4.5	85.7	4.37	81.5
	Sensitivity	92.9	85.7	4.25	85.7	4.14	78.6
	Min. important diff.	92.9	88.9	4.04	71.4	3.96	67.9
	Floor and ceiling	89.3	96.2	3.54	53.6^a^	3.39	46.4^a^
Feasibility	Interpretability	100.0	89.3	4.21	82.1	4.18	82.1
	Familiarity required	78.6	95.7	3.79	71.4	3.75	71.4
	Scoring complexity	92.6	96.2	3.75	57.1^a^	3.86	60.7^a^
	Completion complexity	85.7	96.3	3.54	57.1^a^	3.64	60.7^a^
	Cost	89.3	88.9	3.61	64.3^a^	3.75	67.9
	Duration	92.9	100.0	3.75	67.9	3.93	75.0

Q1 refers to question one and so forth

^a^Consensus not reached on the question for the corresponding item

Three main actions were proposed by the steering committee based on round 1 results and participant feedback. First, a definition of ‘test quality’ was provided for round 2. Quality was defined as ‘the standard of something as measured against other things of a similar kind; the degree of excellence of something’ [40] and was included in round 2 of the questionnaire. Second, the uniform use of ‘exercise and sport sciences’ nomenclature was introduced into definitions and examples to help overcome instances of perceived ambiguity. Third, there were discrepancies in that some items achieved consensus for all questions whereas others did not; written participant feedback expressed that the relevance of certain measurement properties in a test may depend on the context of its use (i.e. laboratory vs. applied settings). Thus, it was determined that the development of level 1 and level 2 levels be used to distinguish between these items. Level 1 items were defined as those achieving group consensus (>67%) in all four questions, and therefore by inference were deemed essential for consideration when selecting or developing any exercise and sport science performance test. Level 2 items included those achieving partial consensus across the four questions. Specifically, this meant that the item was required to have achieved consensus in at least two of the four questions. For example, a level 2 item may have reached consensus on its definition and terminology, however, may not have reached consensus on the Likert-based importance and/or attitude questions. These items were deemed relevant for consideration in some cases; for example, depending on the test’s intended use (i.e. laboratory based or in the field).

Despite achieving consensus on terminology and definitions, the steering committee nonetheless recommended a number of minor revisions for consideration based on written participant feedback. The majority of these related to changes to wording of definitions; however, two additional recommendations relating to items were also proposed. First, it was accepted that minor differences existed between the corresponding definitions for responsiveness and sensitivity to change. Specifically, sensitivity to change referred to ‘the ability of test to detect change, regardless of noise, relevance or meaningfulness’ and responsiveness ‘the ability of a test to detect worthwhile and ‘real’ improvements over time’. However, in the interest of usability, the two items were consolidated as a single item for the checklist (see final definition in Table 2). Second, it was apparent that a number of terms existed in the literature were interchangeable in their use and meaning. In addition to the initially proposed ‘minimum important difference’, this also included ‘smallest worthwhile change’ and ‘smallest worthwhile difference’. Consequently, it was recommended that these also be consolidated into a single item (minimum important difference/smallest worthwhile change).

**Table 2:** Final list of items ranked by level; corresponding definitions are also included

	Item	Definition
Level 1	Re-test reliability	The consistency of performers(s) results over repeated rounds of testing conducted over a period of typically days or weeks. This represents the change in a participant’s results between repeated tests due to both systematic and random error, rather than true changes in performance [27, 36, 46]
	Intra-rater	The agreement (consistency) among two or more trials administered or scored by the same rater [4, 47]
	Inter-rater	The level of agreement (consistency) between assessments of the same performance when undertaken by two or more raters [4, 46, 47]
	Content validity	How well a specific test measures that which it intends to measure [4, 27]
	Discriminant validity	The extent to which results from a test relate to results on another test which measures a different construct (i.e., the ability to discriminate between dissimilar constructs) [42, 48, 49]
	Responsiveness/sensitivity to change	The ability of a test to detect worthwhile and ‘real’ improvements over time (e.g., between an initial bout of testing and subsequent rounds) [42, 50–54]
	MID/SWC	The smallest change or difference in a test result that is considered practically meaningful or important [55–58]
	Interpretability	The degree to which practical meaning can be assigned to a test result or change in result [25, 28]
	Familiarity required	The need to undertake a test familiarisation session with all participants prior to main testing in order to reduce or eliminate learning or reactivity effects [4]
	Duration	Expected and/or actual duration of the testing protocol [59, 60]
Level 2	Stability	The consistency of performer(s) results over repeated rounds of testing conducted over a period of months or years [40, 42, 61, 62]
	Internal consistency	The degree of inter-relatedness among test components that intend to measure the same construct/characteristic [28]
	Convergent validity	The extent to which results from tests that theoretically should be related to each other are, in fact, related to each other [42, 49]
	Concurrent validity	The extent to which the test relates to an alternate, previously validated measure of the same construct administered at the same time [42, 63]
	Predictive validity	The extent to which the test relates to a previously validated measure of a theoretically similar construct, administered at a future point in time [42, 63]
	Floor and ceiling effects	The ability of a test to distinguish between individuals at the lower and upper extremities of performance (i.e., ability to distinguish between high results (ceiling effect) and low results (floor effect)) [28, 64]
	Scoring complexity	The ease with which a test can be conducted and scored in a practical setting by the test administrator [65, 66]
	Completion complexity	The ease with which a test can be completed by a participant [65–67]
	Cost	The total amount of resources required for test administration including equipment, time, and administrator expertise/experience [25]

Reference support for each definition has also been provided

*MID* minimum important difference, *SWC* smallest worthwhile change

### Round 2

**Fig. 2:**
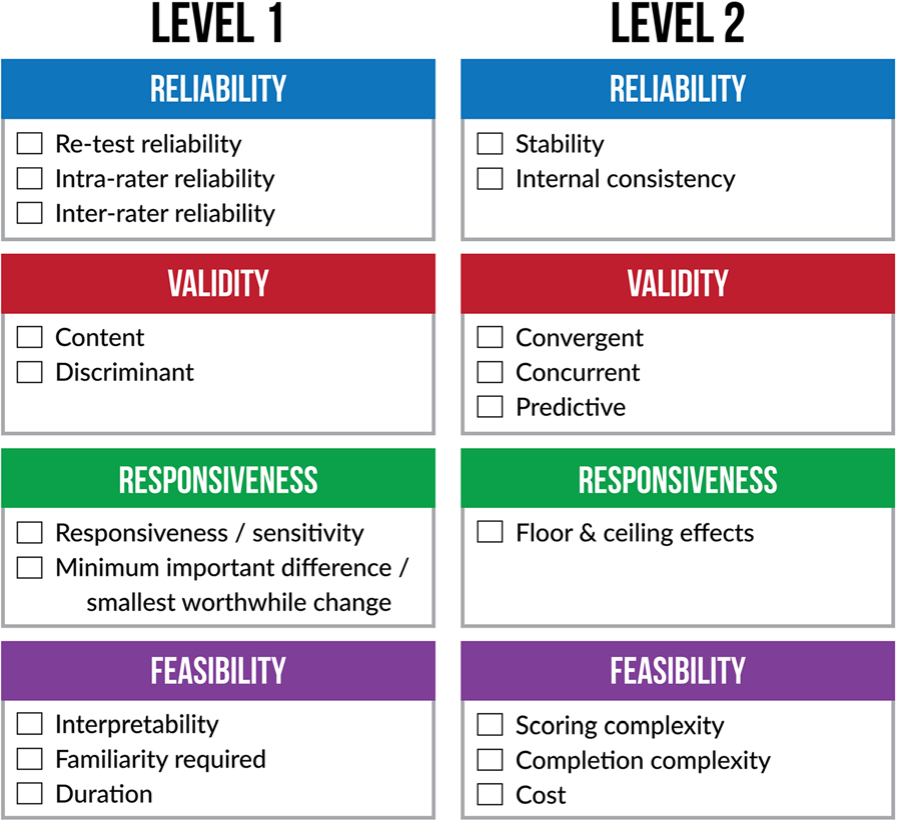
Final taxonomy displaying the 19 level 1 and 2 items important for consideration in evaluating an exercise and sport science performance test

Of the 28 respondents participating in round 1, 20 (71%) also completed round 2 of the questionnaire. This consisted of eight academics, eight sport scientists and four clinical exercise scientists. This was slightly lower than the typically expected 75% retention rate [28] seen in similar studies. Results from the second round revealed that all three main recommendations by the steering committee achieved consensus, along with the minor revisions relating to terminology and definitions. Final definitions and terminology are shown in Table 2, along with relevant reference support. Figure 2 represents a taxonomy of the final list of items.

A checklist derived from the findings of the Delphi questionnaire (at the completion of round 2) has been included as Table 3. The checklist can be implemented by users to record information relating to the measurement properties and feasibility characteristics of a given test, using existing results in the literature. Results from users’ testing on a sample population of interest can also be documented.

**Table 3:**
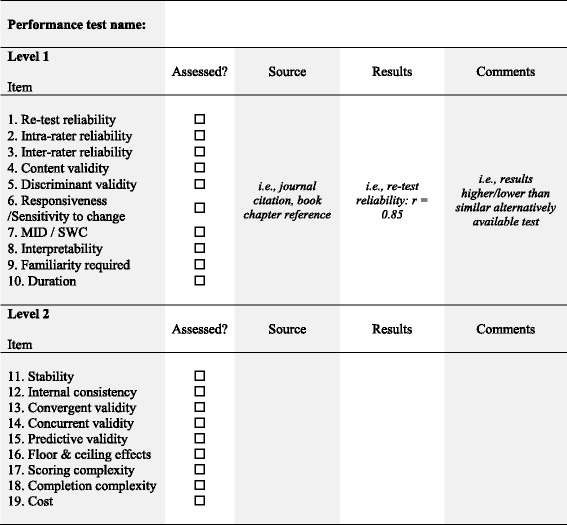
User checklist based on the final results of the Delphi study

All items achieving consensus in the questionnaire are included under the respective ‘level 1’ or ‘level 2’ categories. The user can list previous findings relating the measurement properties and feasibility of a test and/or record their own results

## Discussion

This study primarily aimed to obtain subject matter expert consensus on which measurement and feasibility properties should be considered for performance tests used in the exercise and sport sciences, along with their terminology and definitions. Ten items, including re-test reliability, content validity and responsiveness were considered essential by respondents. A further ten, including stability, predictive validity and concurrent validity, though recognised as important, were considered more context-specific. A secondary aim was to develop a checklist of the agreed upon properties which can inform performance test development or selection.

It was notable that all of the 20 items originally proposed in the first round of the questionnaire were accepted at some level. This suggests that experienced practitioners and academics in the exercise and sport sciences have an appreciation for the importance of measurement quality, but also that there are many components that come together to make a ‘high-quality measure’. The findings also demonstrate that the list was comprehensive, particularly as no additional items were suggested for inclusion by any of the participants. Specifically, commonly reported measurement properties such as re-test reliability, discriminant validity and responsiveness were all included as relevant items based on the final results, thereby confirming their importance for consideration when using a performance test. Based on these results, it would appear that these items be considered by researchers and practitioners alike in a variety of contexts. Measurement properties such as stability and concurrent validity, whilst included in the framework as level 2 items may not necessarily be relevant however under all circumstances. It is worth noting here that the likelihood of a given test displaying an appropriate level of each of these properties will depend largely on the user’s ability to administer it appropriately. Despite these conclusive findings in the participant sample, an increased number of participants from each of the three types of subject matter experts may have allowed for the investigation of whether statistical differences in the responses of these three subgroups existed and more generalisable results overall.

Comparison of the findings of this study also revealed some similarities with work undertaken in other disciplines. Previous checklists developed from research undertaken in the COSMIN project (used in health-related patient-reported test outcomes) also included measurement properties such as reliability, content validity, criterion-related validity, responsiveness and interpretability [28, 41]. The current findings also build additionally on previous work undertaken in exercise and sport science that has espoused the importance of many of the measurement properties included here [1, 4, 15, 16]. Further, in addition to ‘traditional’ measurement properties, this study also considered often overlooked items relating to feasibility in performance testing, which may be particularly important for users working in field environments. Whilst not considered measurement properties per se, items such as test duration, cost and complexity of completion were all deemed important considerations based on results of the current study.

The development of level 1 and level 2 criteria in this study represents a novel addition to previous work from other disciplines. Specifically, these criteria provide the user with flexibility in application of the findings. This is particularly useful as the relative importance of any item may differ depending on the intended use of, or context for, the test [27]. For example, the costs of administering a test may be a critical factor if financial resources are limited, but this may not be a constraint in all settings. Similarly, convergent validity may not be assessable in scenarios whereby a similar measure for comparison is not available.

The development of the checklist based on the findings from this study represents the main practical application of this work. The checklist consists of the 19 level 1 and level 2 criteria from the Delphi questionnaire, which can be used to assess an existing or newly developed performance test. Specifically, when selecting a test for implementation, the user can directly assess its quality based on existing results reported in the literature. These results can be recorded and easily compared against different test options or with newly developed alternative. The checklist also allows for the user to add their own testing results to compare directly with previous findings. This is important because although a test may display appropriate measurement properties and feasibility in one setting, this does not guarantee the same results when applied to a new scenario or population [25, 42]. It is hoped that this feature of the checklist prompts users to undertake their own measurement property and feasibility assessments when using a performance test.

Some limitations of the study should also be stated. The Delphi approach has been criticised due to its potential for researcher bias, its potential issues in achieving appropriate expert selection and has also been considered a restrictive communication method [43]. Further, the authors also acknowledge that the use of a face-to-face method (whilst difficult to facilitate) may have elicited different results to those seen here. Also, participants involved in the Delphi questionnaire were all of a single nationality and an even distribution from each of the three sub-groups was also noted. This may have meant that consensus was easier to achieve, given participants may have had similar conditions in their work environments and also experienced similar socio-cultural norms. There is a potential that engaging an international sample or a different sampling procedure altogether may have elicited different results to those observed here. Further, it is worth noting that the sample was recruited based on their expertise in sport and exercise rather than in measurement. As such, results may have differed somewhat to one that included statisticians or measurement experts.

In addition to addressing some of these limitations, future work in this area may also focus on the development of a user manual to be used as a supplement to the checklist. This manual could include specific practical examples of each item in order to increase the interpretability and increase the practical utility of the checklist for a wider user population. This may also allow for wider dissemination of the checklist to non-academic audiences. Further work may also look to evaluate the properties of the checklist itself. For instance, an evaluation of the uptake of the checklist after a period of time post-implementation may allow for identification of areas in need of further development. The measurement properties of the checklist itself are also still to be determined. For instance, the inter-rater reliability of user implementation of the checklist to rate particular tests may represent an appropriate starting point [36]. Follow-up studies may also look to determine the most appropriate statistical methods available in order to evaluate each item included in the checklist. This would serve to define the actual quantitative quality criteria relating to each item. For instance, in the case of a specific validity item, a minimum level of a particular statistical measure (i.e. correlation statistic) may be determined in order to provide a more specific representation of test quality. This approach, already undertaken in other disciplines [44, 45], could be a valuable addition to exercise and sport science research and practice.

## Conclusions

The aim of the current study was to obtain subject matter expert consensus on which measurement and feasibility properties should be considered for exercise and sport science performance tests. Respondents agreed with the terminology and definitions for all 20 items proposed. Traditional measurement properties such as re-test reliability, content validity and responsiveness were considered essential (level 1) by respondents. Items such as stability, predictive validity and concurrent validity were considered to be more context-specific (level 2) in their application. Establishing a two-level hierarchy for measurement properties is a step-forward in the consensus literature, building on previous research in medicine and health. The checklist developed from the results should serve as a prompt for researchers and practitioners to overtly consider measurement properties in their exercise and sports science practice. Evaluating the implementation, use and measurement properties of the checklist itself is an obvious next step to further assist rigorous and transferable exercise and sports science research and practice.

## Abbreviations

COSMIN: Consensus-based standards for the selection of health measurement instruments
RR: Response rate
